# The Effects of Long-term Use of Nitrogen-containing Bisphosphonates on Fracture Healing

**DOI:** 10.7759/cureus.4307

**Published:** 2019-03-25

**Authors:** Dimitrios Begkas, Alexandros Pastroudis, Panagiotis Touzopoulos, Nikolaos G Markeas, Stamatios-Theodoros Chatzopoulos

**Affiliations:** 1 Orthopaedics, Asclepieion Voulas General Hospital, Athens, GRC; 2 Orthopaedics, University General Hospital of Alexandroupoli, Alexandroupoli, GRC; 3 Orthopaedics, Athens Children's Hospital, Athens, GRC

**Keywords:** fracture, fracture healing, bisphosphonates, nonunion, osteoporosis

## Abstract

Nitrogen-containing bisphosphonates (N-BPs) are pharmaceutical agents that have been used for many years to treat osteoporosis, multiple myeloma, Paget's disease, metastatic bone disease, and a variety of other diseases in which bone mineral density is reduced. Given that N-BPs inhibit bone resorption, an important stage in the fracture healing process, they have been extensively studied in preclinical models for their activity. In animal models, treatment with N-BPs is associated with a larger callus formation in fracture area and delay in remodeling from primary woven bone to lamellar bone, but there is no delay in formation of the fracture callus. In humans, all existing evidence suggest that initiating treatment with N-BPs, after upper and lower limb fractures, does not appear to have a significant effect on fracture healing. Rarely, patients with long-term use of N-BPs may develop “atypical fractures” and delay in their healing. Therefore, this clinical condition is not fully understood and many questions remain unanswered. Similarly, there are few studies about the benefits of stopping a long-term treatment with them, if a fracture occurs. Although most studies support that chronic N-BP therapy may lead to fracture healing delay, this is not fully documented. On the other hand, there are studies that are in complete disagreement with them. All of the above suggest that there is a need for more detailed future research into larger patient populations and different types of fractures, with sufficient data on the type, dosage, route and duration of administration of N-BPs, and the control methods of fracture healing, in order to have a safe final conclusion on the effect of their long-term administration in this highly complex process.

## Introduction and background

Nitrogen-containing bisphosphonates (N-BPs) are pharmaceutical agents commonly used for the treatment of various metabolic bone disorders, including osteoporosis, Paget's disease, metastatic bone disease, etc. Their use in the treatment of osteoporosis has been shown to be extremely effective, which leads to an increase in bone mass, reduces the rate of bone turnover, and ultimately reduces the incidence of fractures [1].

N-BPs show high calcium selectivity and in the body are concentrated in the skeleton at the sites where bone remodeling occurs. They are incorporated into the newly formed bone during the anabolic phase, via their attachment to hydroxyapatite salts, where they remain inactive. When the bisphosphonate-containing bone is absorbed, the bisphosphonates are released into the acidic environment of the Howship’s lacunae and they are absorbed by the osteoclasts. The mechanism of action of the N-BPs involves the inhibition of farnesyl pyrophosphate synthase (FPPS), a key enzyme in the mevalonic acid pathway. This results in cytoskeletal changes in osteoclasts, inhibition of their activity and apoptosis [2]. The ultimate and most important result is the reduction in bone resorption. Because of the long-term suppression of bone catabolism, patients receiving N-BPs show a constant reinforcement of the mineralized bone tissue. It was initially considered that bisphosphonates inhibit only the absorption of the existing bone, thus retaining its architecture. However, it was further revealed that they also allow for enhanced or prolonged secondary mineralization [3].

Bisphosphonates are buried in the bones and remain in the body for a long time after cessation of treatment. The estimated half-life for their removal from the skeleton is up to 10 years. This is also evidenced by the observation of detectable levels of pamidronate in patients' urine eight years after cessation of treatment [4]. Since bone resorption from osteoclasts is a key component of bone fracture healing, concerns have been raised about the use of bisphosphonates and possible inhibition of the fracture healing process, especially after their long-term use and accumulation in the skeleton. In recent years, the number of osteoporotic patients undergoing long-term N-BP therapy has been increasing steadily. So the question is whether the same applies to the possible negative effects that these drugs may have on the skeleton and whether they can affect the healing process of bones after fractures. Of particular concern are questions such as:

1. Is it possible that the administration of N-BPs inhibits bone anabolism?

2. Can N-BPs suppress the early stages of endochondral fracture healing?

3. Do N-BPs disturb the process of bone remodeling during the final phase of fracture healing?

In the present study, we will try to answer the above questions by presenting all the references in the international literature, including data from animal model surveys, as well as human studies, with the main aim of arriving at a final conclusion on the effects of long-term use of N-BPs on fracture healing.

## Review

Effect of administration of N-BPs on bone anabolism

Administration of N-BPs causes temporary decoupling between bone anabolism and catabolism, resulting in reduced catabolism without a corresponding reduction in anabolism. However, their chronic administration may also lead to the suppression of anabolism. Although many in vitro studies have shown a mild positive effect of N-BPs on the proliferation and differentiation of osteoblasts [5], this finding is in conflict with many other animal and clinical studies suggesting that treatment with them may actually reduce the bone formation [6-8]. In an effort to better understand the in vivo effects of N-BPs on bone anabolism, three groups of rats were subjected for more than five months to daily application of risedronate (0.1, 1.0 or 10 μg/kg) in the first group, alendronate (0.05, 0.5 or 5.0 μg/kg) in the second group and normal serum (0.9% NaCl) in the third group. The Mineral Apposition Rate (MAR) in the femoral and tibial bones was significantly reduced in the first two groups (26%-36% for alendronate, 22%-29% for risedronate) [8]. These data suggest the possibility that chronic treatment with N-BPs can significantly reduce bone anabolism, although the exact mechanism for this remains unclear. In contrast, in a bone biopsy study in patients undergoing annual treatment with zoledronic acid for osteoporosis, MAR remained stable [9].

In another study, nine patients who had fractures during long-term treatment with alendronate due to osteoporosis (10 mg/day or 70 mg/week) were reported. Delayed fracture union was observed in six cases, ranging from three months to two years after the fracture [7]. Bone biopsies from all patients revealed the complete absence of double marking in normal elderly controls, while bone formation rates declined up to 100 times in these patients. In four cases, delayed union or failure of fracture healing was also reported after discontinuation of treatment. The authors of this study consider that the substantial reduction in the bone turnover cycle of these patients was manifested as a reduced anabolic response that delayed fracture healing. However, it cannot be confirmed that alendronate treatment was the only determinant factor that led to this.

On the contrary, in a study of closed fractures in children with osteogenesis imperfecta under chronic treatment with high doses of pamidronate, there was no delay in fracture healing [10]. On the other hand, in patients with osteotomies while undergoing pamidronate treatment, there were significant delays in their fracture healing. This difference can be attributed to mechanical stimuli during limb loading, or to reduced anabolic activity due to soft tissue damage after surgery. It is likely that this phenomenon can be attributed to the disease itself, as there are studies showing differences in osteoblastic response to alendronate between patients with osteogenesis imperfecta and control groups [11]. A more recent study of similar patients with osteogenesis imperfecta confirmed that pamidronate showed no effect in the process of fracture healing after closed treatment [12].

Perhaps the most relevant clinical evidence is derived from a zoledronic acid test in patients with hip fractures [13]. In this study, 1,065 patients with hip fractures were randomized to receive zoledronic acid within 90 days after the fracture and 1062 to receive placebo. Subsequent injections were given annually. The results showed significantly reduced rates of subsequent clinical fractures and mortality improvements. An important observation was that there was no difference in the clinical delay of fracture healing between zoledronic acid (3.2%) and placebo (2.7%) (P = 0.61). It is also important to note that this study was based on post-fracture follow-up of the patients for symptomatic hip pain, without being subjected to systemic x-ray tests in order to control the possibility of delayed fracture union. However, the absence of an impressive increase in cases of delayed union provides some assurance that the use of zoledronic acid may reduce the likelihood of future fractures without interfering healing of the initial fracture.

Effect of administration of N-BPs on endochondral ossification

Until recently, osteoclasts were considered to be the main cell type responsible for cartilage removal during endochondral fracture healing. This had raised concerns about whether N-BPs interfere with the replacement of the soft cartilaginous callus by a hard callus of bone tissue and if they lead to prolonged mechanical instability and delayed fracture union. Under these circumstances, it would be preferable to discontinue treatment of osteoporotic patients with fractures. However, there is evidence from laboratory and clinical studies showing that this is not happening.

In a study conducted primarily to control osteoclastic resorption in patients treated with N-BPs, osteoclasts have been shown to be unnecessary for the early stages of soft callus replacement. In experiments performed in rats with femoral fractures treated with pamidronate (open fractures) and zoledronic acid (closed fractures), during their radiological follow-up, fracture healing did not appear to be affected by the use of these drugs [14-15]. In addition, as an alternative model of osteoclast dysfunction induced by N-BPs, endochondral fracture healing was examined in an osteoporotic rat (where osteoclasts are non-functional). As with the other animals treated with N-BPs, the initial stages of fracture healing were also progressed normally [16].

The above data suggest that the functional activity of osteoclasts may be superfluous for the generalized process of endochondral ossification. However, there is a study which demonstrates that inhibition of osteoclast function does not have adverse effects on the epiphyseal plates of long bones in mice [17]. The authors of the study eliminated osteoclast function from endochondral ossification by using clodronate in two osteoclast deficient mutant mouse models (the c-fos knockout mouse and the osteopetrotic [op/op] mouse). Based on these findings, it is reasonable to assume that cells that compensate for osteoclasts to remove cartilage from epiphyseal plates may also compensate for the early stages of endochondral fracture healing.

Complete inhibition of fracture healing has also been observed in a rabbit model with embryonic bone abnormalities after administration of high topical doses of pamidronate [18]. Two or three milligrams of pamidronate were applied via a poly L-lactide-co-glycolide (PLGA) carrier. These doses of pamidronate resulted in significantly reduced bone formation within two weeks and apparent avascular necrosis at later stages. In particular, as a fracture healing model, this case does not reflect the physiological endochondral processes occurring in the long bones. It is therefore unlikely that the lack of cartilage removal hides the poor fracture healing caused by pamidronate. Instead, it is more likely to happen through a combination of antiangiogenetic metalloproteinases (MMPs) and local cytotoxic effects caused by concomitant administration of pamidronate. Unlike clinical dosing, where bisphosphonates rapidly bind to bone minerals and remain in tissue fluids for only a few hours, this polymer dosing method allows continuous concentration of pamidronate and therefore its effect on other cells than from osteoclasts. In a distraction osteogenesis study in rabbits, where lower doses produced benefit, a high dose of alendronate (75 μg/kg weekly) inhibited fracture healing [19].

From the results of the above studies, it seems that the application of N-BPs can produce certain advantages, which have to do with the transient preservation of the early bone scaffold and the increase in the size and strength of the newly formed bone during the endochondral fracture healing (due to neutralization of the osteoclastic activity). In very high doses, however, they can actually prevent the anabolic response and therefore such dosages should be avoided.

Effect of N-BPs administration on bone remodeling during the final fracture healing phase

Remodeling of the hard bone callus that takes place through the endochondral processes is the final stage of fracture healing. By absorbing of primary woven bone and its replacement with mature lamellar bone, the volume of callus decreases and the fracture area receives again its original structural shape. Osteoclastic resorption is undoubtedly necessary for this process, and numerous studies document bone callus remodeling due to concomitant administration of N-BPs [20-21]. Until the remodeling phase, the maintenance of the primary woven bone is beneficial as it improves the mechanical strength of the callus and therefore its resistance to a possible refracture. However, after the completion of fracture healing, the maintenance of primary callus can be detrimental due to the inferior mechanical properties of the material, compared to those of the mature lamellar bone that ultimately replaces it [22].

Morphologically, prolonged treatment with N-BPs may lead to failure of mature lamellar bone or new cortex formation around callus and to the maintenance of the primary woven bone tissue. Incadronate treatment in rats at doses of 0.01 or 0.1 mg/kg, three times weekly for two weeks prior to fracture surgery and then up to 16 weeks postoperatively, caused an extensive increase in the callus size [23]. Even at this late stage of fracture healing, the newly formed bone was not remodeled and did not form a cortical envelope, as was observed in a group of mice that had not received incadronate. Other experiments, where N-BPs were applied to rats, showed similar results [24]. Regarding overall mechanical strength, a higher callus volume in animal models treated with N-BPs can generally compensate for the lack of inherent mechanical properties of the bone, yielding similar or increased resistance to a possible refracture. However, the normalization of the total callus volume demonstrates that the primary woven bone has inferior material properties in relation to the remodeled lamellar bone [25]. Thus, a prolonged or indefinite delay in bone callus remodeling can be considered as an adverse outcome that could affect its resistance to fatigue or the energy required to cause a new fracture.

Based on the data showing that a single application of N-BPs has the ability to produce a stronger primary woven bone callus, with the ability to further remodel, we understand that it may be feasible to be reduced the frequency of their therapeutic doses, so as to be minimized their effects on the hard callus remodeling [14-15,25]. Further studies will be required for the development of optimal dosages and the implementation of these concepts in clinical practice.

Results of long-term application of N-BPs οn fracture healing

Preclinical Studies in Animal Models

A variety of animal models has been used to examine the effects of N-BPs on fracture healing, including mice, rats, rabbits, dogs, and sheep. Using these models, the effects of long-term N-BP application on the secondary fracture healing have been extensively examined and their results are particularly convincing, unlike those in primary fracture healing where they have been less studied. Overall, these secondary fracture healing studies suggest that chronic application of N-BPs appear to reduce callus remodeling, while increasing fracture bridging and/or maintaining of cancellous bone structures within the callus [15,26]. The main conclusion is that long-term treatment with N-BPs does not prevent the formation of the callus itself, but there is a delay in its conversion from woven and lamellar bone tissue [14, 27-28].

Results οn secondary fracture healing: Fu et al. studied the properties of fracture calluses in ovariectomized rats. They noticed that after long-term administration of alendronate, the callus formed was larger. However, despite observing a delayed transformation of the woven bone into mature lamellar bone in the intervention group, the mechanical properties of callus were similar to rats in the control group [28]. Manabe et al. presented similar results using ibandronate. In this study, the authors noted that the prolongation of the time interval between drug doses could reduce the delay in the transformation of the woven bone of the callus into mature lamellar bone tissue [29].

Kidd et al. studied the effects of high and low doses of risedronate on the healing of ulnar stress fractures in rats. Those who received the highest dose (1.0 mg/kg, twice the normal dose of osteoporosis treatment) were found to have a delay in fracture healing. In particular, a decrease in bone resorption and in the formation of new bone along the fracture line was observed at six and 10 weeks after the fracture. This delay was not observed in rats treated with low dose (0.1 mg/kg). Irrespective of the doses of risedronate used, no interference was observed in the callus formation [30]. Sloan et al. had similar results in a similar study in which alendronate was applied [31].

Yu et al. observed a delay in the early phases of fracture healing in mice receiving zoledronic acid, such as delay in cartilage hypertrophy, in angiogenesis and later in callus remodeling. This effect was stronger, in mandible fractures than those of the tibia [32]. It should be noted that these results do not agree with those of similar studies in rabbits, where it was observed that the use of zoledronic acid accelerates healing of mandible fractures [33].

Results on primary fracture healing: Primary fracture healing is typically observed with the use of rigid internal fixation, such as in osteosynthesis of transverse diaphyseal fractures of long bones with compression plates and screws. Savaridas et al. examined the effect of previous Ibandronate application on osteotomies of rat bones that were stabilized by compression plates and screws. They found attenuation of fracture healing progression, reduction in mean failure fatigue as assessed in four-point bending tests and reduction of bone density at the site of osteotomy in the treated rats rather than the controls. Finally, the presence of cartilaginous and undifferentiated mesenchymal tissue was observed at the site of osteotomy [34].

Studies in Humans

N-BPs are the most commonly used drugs in humans for the treatment of osteoporosis. It has been observed that in most preclinical model studies the callus formed is larger, but fracture healing is not affected by their application [35]. Xue et al., in a meta-analysis of eight randomized controlled trials about fracture healing when N-BPs are applied after the fracture, they concluded that there was no delay in callus formation or fracture healing [36]. In general, the observation that N-BPs do not affect fracture healing is mainly found in studies focused on specific types of fractures, but this is not a universal observation. At the upper limb, the use of N-BPs has been studied for many different types of fractures, where most authors report no significant healing delay, but there have been reports of an increased rate of nonunions [37]. Conversely, fracture healing in patients that were already under long-term treatment with N-BPs at the time of fracture is reported that was delayed, but there was no difference in the rate of nonunions [37]. In another study, the long-term use of N-BPs prior to fractures of the distal radius resulted in a small delay (less than a week) in their healing, but in a separate study, early N-BP application following fractures of the distal radius did not have the effect of delaying their healing [38-39].

Schindeler et al., in a broad retrospective study of the effects of N-BPs on fracture healing, consider that their application after fractures is safe at the usual dosages used to treat osteoporosis. However, for patients who have already undergone long-term N-BP treatment (more than three years), they suggest stopping them for as long as the fracture is healing, without recognizing any particular risk of new osteoporotic fractures within that time. This prevents the production of an oversized callus, while the remodeling process is not disturbed [40].

Key cases where many authors report a delay in endochondral fracture healing are in previous long-term use of N-BPs, as well as in the treatment of atypical femoral fractures associated with N-BPs [41-42]. However, there are studies in the literature, where are not observed serious effects of chronic N-BP use on fracture healing. Molvic and Khan, in a systematic review [43], investigated (in six studies) the association between long-term application of N-BPs and the average duration of fracture healing. One of these studies included fractures of the distal radius, and the rest involved fractures of the femur. The study on distal radius fractures included 43 cases with a history of chronic N-BP use prior to them. The mean treatment time with N-BPs was 25 months (SD 21 months, range 1-120 months). Although their use seemed to be associated with longer fracture healing time, Molvic and Khan paid particular attention to the fact that the authors of the study avoided coming to that conclusion. As about the studies for the femoral fractures, there was no correlation between the duration of the treatment and the time of fracture healing. Only two studies contained details of long-term treatment with N-BPs prior to fractures and the number of cases of nonunions that occurred. Since there was only one nonunion case in each of these studies, it was not possible to be investigated their association with the chronic uptake of N-BPs [43]. Ng et al. noticed a slight healing delay in upper limb fractures following long-term application of N-BPs but no difference in the incidence of nonunions [37].

In contrast to the above, Armamento-Villareal et al., in their histological study of fractures in various bones, reported on extensive suppression of bone turnover in patients under long-term N-BP therapy [44]. This was a retrospective cohort study examining the low energy cortical fractures of 15 patients who had received N-BPs for an average of 5.7 years (range 2-10 years). Fourteen patients received alendronate and one risedronate. The fractures were located on the femoral shaft, the ribs, the metatarsals, the pelvis, the fibula, and the ankle. It was found that bone biopsies of patients with suppression of bone turnover (10 patients, 67%) had a 6.5 year mean duration of N-BP use, while in bone biopsies of patients with normal bone turnover (five patients, 33%), the mean time of N-BP use was 3.9 years (p = 0.02). The authors also found that there were no significant differences in age, body mass index (BMI), bone mineral density (BMD), calcium and vitamin D intake, serum calcium, parathyroid hormone, and 25 hydroxyvitamin D, between patients with normal and suppressed bone turnover.

As mentioned above, a key case in which many authors report a delay in fracture healing is the treatment of atypical femoral fractures associated with long-term use N-BPs. Unfortunately, there has been observed a healing delay in 26% of cases of these rare fractures [42].

Egol et al. treated 33 patients with 41 atypical, low energy femoral fractures by intramedullary nailing. All patients received chronic N-BP therapy, averaging 8.8 years (range, 5-20 years) before the fracture. Although a delay was observed, finally fracture healing was achieved in the majority of them [45].

Prasarn et al. reviewed retrospectively 43 patients with atypical femoral fractures associated with long-term use of N-BPs and 20 patients with similar fractures that were not receiving N-BP therapy. Similar implants were used in both groups, but in the group of those who had received N-BPs, a greater number of fracture healing stimulators was used. Despite the low rates of other risk factors and the high use of biological stimulators, patients treated with N-BPs exhibited more complications (material breakage, intraoperative fractures) and delayed healing [46].

Weil et al. treated 15 patients that suffered from 17 atypical femoral fractures associated with long-term N-BP therapy (mean duration of treatment 7.8 years). The percentage of healed fractures was 54%, while 46% underwent new surgery (nail dynamization, nail replacement, or plate and screw fixation). In the end, all the fractures healed. The authors concluded that the use of intramedullary nailing in this type of femoral fractures presents a very high failure rate and often requires revision surgery [47].

Discussion

Osteoclasts play a key role in bone remodeling and metabolism. This raises concerns about the use of N-BPs on fracture healing. However, evidence suggests that the strategic management of osteoclastic activity may be neutral or even advantageous regarding the end result. Studies suggest that chronic administration of N-BPs may gradually reduce bone formation. This reduction in anabolism may affect the healing of bone fractures, particularly in the most serious cases where a significant anabolic response is required [10]. Thus, discontinuation of N-BPs (drug holiday), in order to allow recovery of the natural endogenous anabolic response, may provide benefits to patients undergoing long-term treatment of osteoporosis with them [48].

While osteoclasts are often assumed to be essential for the early stages of endochondral fracture healing, numerous studies have shown that N-BPs do not adversely affect this process. In addition, by suppressing early bone catabolism, they can maximize the size and strength of the original callus and in the future to reduce the possibility of refracture [40].

Some studies have demonstrated that prolonged daily application of N-BPs prevents the transformation of woven primary bone in normal lamellar bone. While this presents itself as the main problem, the large woven bone callus still exhibits considerable mechanical strength [28]. However, most authors believe that remodeling would be beneficial and that exploiting the early benefits of increasing of the callus size can be achieved by a single N-BP application, while retaining the ability of later remodeling [14-15,25].

In studies performed about fracture healing in N-BP treated animal models, virtually all species studied exhibited greater callus formation and delayed remodeling of primary woven bone to mature lamellar bone tissue. Despite the deviation from the normal healing pattern, this largest size of the observed callus is usually reported to be mechanically equivalent but not superior to what was found in fracture healing of animal control groups. An increase in biomechanical strength may be due to the preservation of the trabecular structure within the callus and the increased bridging of the fracture. It should be noted that the time of application of N-BPs in relation to the time of the fractures, as well as their increased dosing regimens in comparison with those used to treat osteoporosis, appear to affect this delay in fracture healing.

Compared to animal models, there are fewer studies on fracture healing in people treated with N-BPs. In all those reported to date, there was no delay in healing of the upper or lower limb fractures when the treatment started immediately after the fracture [36]. In contrast to them, in some patients who had already received long-term treatment and/or suffered from an atypical femoral fracture, there was some delay in the time of endochondral fracture healing [41]. Therefore, some authors believe that the application of N-BPs to patients after fractures (at the usual treatment doses of osteoporosis) is safe. However, in those patients who have undergone long-term N-BP treatment (over three years), they suggest stopping it during fracture healing, without considering that there is an increased risk of new osteoporotic fractures. In this way, they believe that the excessive callus enlargement and the delay in bone remodeling are prevented [40].

## Conclusions

The existing data from international literature studies on delays or complications in the healing of typical fractures of patients under long-term N-BP treatment are inadequate and therefore this clinical condition is not fully understood and many questions remain unanswered. Thus, we suggest that there is a need of more detailed future research into larger patient populations and different types of fractures, with sufficient data on the type, dosage, way and duration of administration of N-BPs, and the control methods of fracture healing, in order to arrive at a safe and final conclusion on the effect of long-term application of N-BPs in this highly complex process.
